# Peptide Receptor Radionuclide Therapy in Advanced Refractory Meningiomas: Efficacy and Toxicity in a Long Follow-up

**DOI:** 10.2967/jnumed.123.266956

**Published:** 2024-09

**Authors:** Stefano Severi, Ilaria Grassi, Alberto Bongiovanni, Silvia Nicolini, Irene Marini, Donatella Arpa, Nicoletta Ranallo, Irene Azzali, Valentina Di Iorio, Anna Sarnelli, Monti Manuela, Elena Amadori, Lucia Fabbri, Daniela Bartolini, Luigino Tosatto, Francesco Di Meco, Lorena Gurrieri, Nada Riva, Luana Calabro, Federica Matteucci, Giovanni Paganelli, Maddalena Sansovini

**Affiliations:** 1 Nuclear Medicine and Radiometabolic Units, IRCCS Istituto Romagnolo per lo Studio dei Tumori “Dino Amadori”, Meldola, Italy;; 2 Osteoncology and Rare Tumor Center, IRCCS Istituto Romagnolo per lo Studio dei Tumori “Dino Amadori”, Meldola, Italy;; 3 Radiotherapy Unit, IRCCS Istituto Romagnolo per lo Studio dei Tumori “Dino Amadori”, Meldola, Emilia Romagna, Italy;; 4 Biostatistics and Clinical Trials, IRCCS Istituto Romagnolo per lo Studio dei Tumori “Dino Amadori”, Meldola, Italy;; 5 Oncological Pharmacy Unit, IRCCS Istituto Romagnolo per lo Studio dei Tumori “Dino Amadori”, Meldola, Emilia Romagna, Italy;; 6 Medical Physics Unit, IRCCS Istituto Romagnolo per lo Studio dei Tumori “Dino Amadori”, Meldola, Italy;; 7 Radiology Unit, IRCCS Istituto Romagnolo per lo Studio dei Tumori “Dino Amadori”, Meldola, Italy;; 8 Pathology Unit, “Maurizio Bufalini” Hospital, Cesena, Italy;; 9 Department of Neurosciences, Neurosurgery Division “M Bufalini” Hospital, Cesena, Emilia Romagna, Italy;; 10 Department of Neurosurgery, Fondazione IRCCS Istituto Neurologico C. Besta, Milan, Italy;; 11 Department of Neurosurgery, Johns Hopkins University, Baltimore, Maryland;; 12 Department of Oncology, University Hospital of Ferrara, Cona, Italy; and; 13 Department of Translational Medicine, University of Ferrara, Ferrara, Italy

**Keywords:** oncology, peptide receptor radionuclide therapy, ^177^Lu, meningiomas

## Abstract

Recurrence of meningiomas after surgery and radiotherapy deserves specific attention because of the lack of active third-line therapies. Somatostatin receptors are usually overexpressed on the cell membrane of meningiomas, and this has led the way to a radionuclide theranostic approach. Diagnoses with ^68^Ga-DOTA-octreotide and peptide receptor radionuclide therapy (PRRT) with ^90^Y/^177^Lu-DOTA-octreotide are currently possible options within experimental protocols or as compassionate use in small patient groups. **Methods:** From October 2009 to October 2021, 42 meningioma patients with radiologic recurrence after standard therapies were treated with ^90^Y-DOTATOC (dosage of 1.1 or 5.5 GBq) or with ^177^Lu-DOTATATE (dosage of 3.7 or 5.5 GBq) in a mean of 4 cycles. All patients showed intense uptake at diagnostic ^68^Ga-DOTATOC PET/CT or in an ^111^In-octreotide scan. **Results:** Of 42 patients treated, 5 patients received ^90^Y-DOTATOC with a cumulative activity of 11.1 GBq and 37 patients received ^177^Lu-DOTATATE with a cumulative activity of 22 GBq. The disease control rate was 57%. With a median follow-up of 63 mo, median progression-free survival was 16 mo, and median overall survival was 36 mo. Retreatment ^177^Lu-PRRT was performed in 6 patients with an administered median activity of 13 GBq in a mean of 5 cycles. With a 75.8-mo follow-up, median progression-free survival and overall survival were 6.5 and 17 mo, respectively. Only 1 patient discontinued the treatment because of grade 3 platelet toxicity. A rapidly transient grade 2 neutropenia was recorded in 1 retreated patient. **Conclusion:** PRRT in patients with advanced meningiomas overexpressing somatostatin receptor 2 was active and well tolerated, showing a 57% disease control rate. Furthermore, PRRT could represent a potential retreatment option. Further studies, also in combination with other treatments, are warranted.

## Visual Abstract

**Figure:**
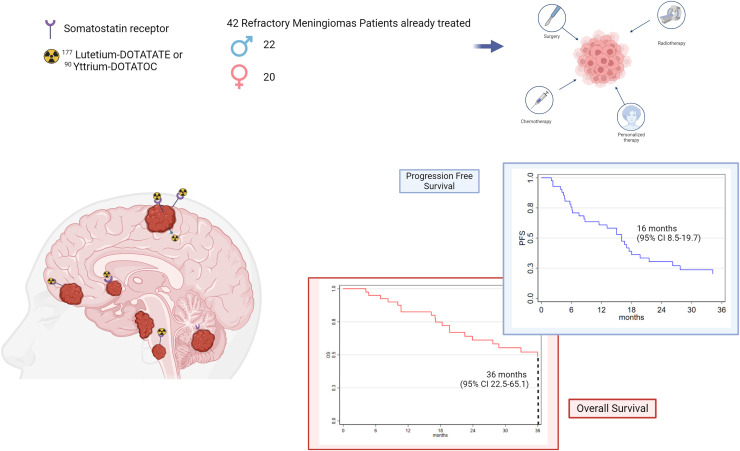

Meningiomas arise from the arachnoid granulation cells in the meninges, which enclose the brain and spinal cord. They are the most common type of all nervous system tumors, accounting for over 30% of all cases, with an incidence of 7.8 cases out of 100,000 (1*,*^2^).

Benign meningiomas make up about 80% of all cases and are classified as typical (World Health Organization grade I). The remaining 20% are either atypical (grade II) or anaplastic (grade III) and are characterized by a more aggressive phenotype and a shorter survival time compared with typical phenotypes. Meningiomas can be intra- or extraaxial neoplasms and are usually asymptomatic. Symptoms, such as seizures, headaches, vision loss, and other neurologic deficits, are related to the size and position of the tumor. Certain familial syndromes and exposure to ionizing radiation can increase the risk of developing meningiomas by 6–10 times (2*,*^3^).

The leading strategies in the management of meningiomas are based on tumor size, position, and grade, with treatment being particularly challenging for skull base tumors because of the presence of vital structures.

Frequently diagnosed as incidental findings in asymptomatic patients, less than 50% of the newly diagnosed meningiomas have surgical indications and may be fully resectable (4).

Nevertheless, surgical resection is the most important treatment for symptomatic meningioma patients to obtain R0 or debulking to decompress the affected areas and reduce symptoms.

Typical meningiomas are observed clinically and radiographically after the initial resection. Radiation therapy is decided on the basis of the meningiomas’ grade and recurrence risk when the tumor could be surgically approached. For surgically untreatable meningiomas, radiotherapy represents a valid alternative to control local growth, but it is not as effective as surgery for symptom relief (5).

Radiotherapy is generally used after surgery for anaplastic meningiomas; however, in cases with atypical tumors, radiotherapy is not always mandatory, and the decision should be considered within a multidisciplinary board (6).

Despite some positive experiences, however, the use of radiotherapy remains the subject of controversial discussions.

At the present time, medical treatments for meningiomas remain largely experimental because of the limited results obtained. Generally, medical treatments are used in cases of recurrent or progressive disease not suitable for surgery or radiotherapy. Although drugs such as bevacizumab, sunitinib, everolimus, and different chemotherapy approaches with temozolomide, irinotecan, imatinib, and others have been tried, there is no clear evidence on the standard of care, and enrollment in clinical trials is recommended in cases of disease progression (7*,*^8^).

There is evidence that a significant percentage of meningioma cells (80%) express somatostatin receptors (SSTRs), making it possible to apply a nuclear medicine theranostic approach with ^68^Ga-DOTA peptide for diagnosis and electrons for therapy with β-emitters such as ^177^Lu or ^90^Y (9–11).

In analogy with experiences in gastroenteropancreatic neuroendocrine tumors, for which a specific radiopharmaceutical with ^177^Lu-DOTATATE is registered (12), peptide receptor radionuclide therapy (PRRT) can also be applied in other neuroendocrine or SSTR2-positive tumors. Furthermore, some published studies have shown a certain effectiveness of PRRT retreatment in neuroendocrine tumors when the positivity of the ^68^Ga-DOTA peptide PET/CT persists at all disease sites (13–15).

Over time an extensive experience has been gained by applying ^90^Y-DOTATOC and ^177^Lu-DOTATATE PRRT to many different histotypes. Despite its proven potential, this option has been scarcely practiced in patients with symptomatic or progressive meningioma lesions (16–21). In the present work, we report the overall outcome for meningioma patients treated with PRRT in our institute.

## MATERIALS AND METHODS

From October 2009 to October 2021, 42 patients with progressive meningiomas were enrolled in various phase II protocols (see additional information below). Initially, we used ^90^Y-DOTATOC on the basis of availability, and later, when ^177^Lu-DOTATATE became available, it was used exclusively.

**FIGURE 1.**
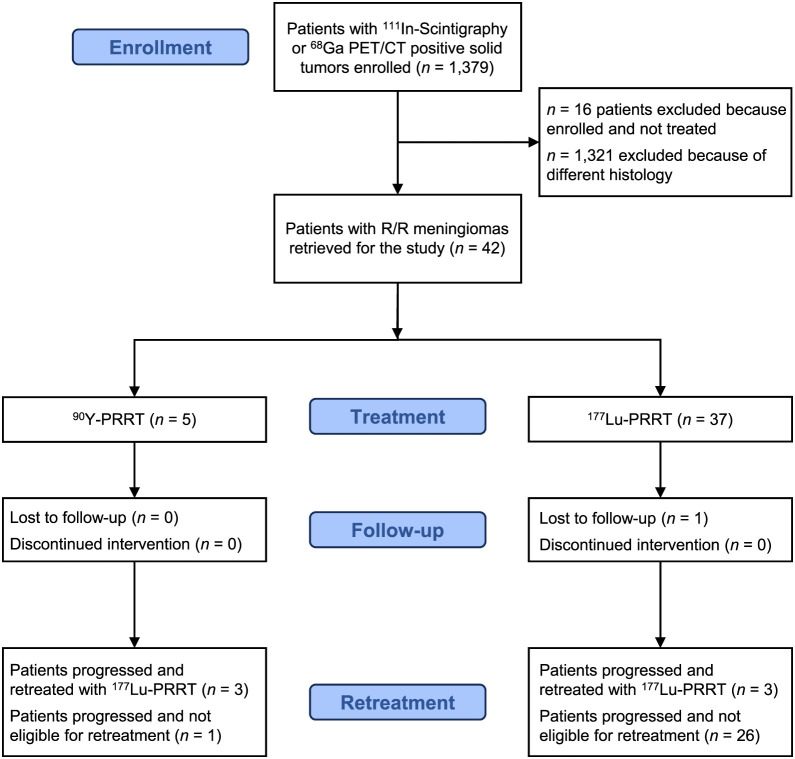
Flow diagram of patients selection and treatment. R/R = recurrent or refractory.

Disease progression was assessed by MRI. The possible inclusion in a clinical trial was decided after a multidisciplinary discussion and approval (Fig. 1). The IRCCS, IRST review board, and AVR Ethical Committee approved all phase II protocols, and all enrolled subjects provided written informed consent. Patients were treated with ^90^Y-DOTATOC at a dosage of 1.1 or 5.5 GBq or with ^177^Lu-DOTATATE at a dosage of 3.7 or 5.5 GBq. In both cases, lower dosages of 1.1 and 3.7 GBq were reserved for patients with bone marrow or kidney toxicity risk factors. Protocols provided 4 to 5 cycles of therapy performed 5–8 wk apart. Six patients had indication for PRRT retreatment performed with ^177^Lu-DOTATATE at a dosage of 3.7 GBq per 4 or 5 cycles repeated 5–8 wk apart. Favorable conditions for this indication were the absence of alternative therapies, an occurrence of symptoms or a fast-progressing disease, a pronounced benefit from prior PRRT, and the lack of G4 or protracted toxicity after the latter.

Patients were pre- and posttreated with standard PRRT amino acid infusions (22). Patients were administered 4 mg of dexamethasone intravenously once a day and 500 mL of 10% mannitol twice a day, the same day and the day after the treatment to lessen the potential of posttherapy edema in the brain lesion. Dexamethasone was also continued with a decreasing dosage for at least 10 d after therapy.

All treated patients had ^111^In-octreoscan and ^68^Ga-DOTATOC PET/CT (SSTR2 scintigraphy) images that were positive with high tracer concentration, with a grade of at least G3 or G4 on the Krenning scale (23) in all lesions, and were classified for grading according to the World Health Organization as detailed.

The tumor burden was classified into 2 different grades: 1–3 lesions were classified as moderate; more than 3 and huge lesions were classified as extended. The masked qualitative evaluation was performed by 2 highly qualified nuclear medicine doctors. In cases of disagreement, the involvement of a third expert aided in achieving a consensus.

Performance status (PS) was defined according to the Eastern Cooperative Group criteria, and patients were excluded if PS was greater than 2. Significant clinical neurologic details, including the existence of neurologic symptoms ranging from mild headaches to neurologic impairments, and the requirement for anticonvulsant or antiedema medication therapy were recorded.

Due to previous experiences with successful PRRT tolerance, the use of modest dosages and in consideration of the patient’s age and general well-being, transient G1 or G2 medullary and kidney toxicities have not been reported in the present article. We decided to point out only the sustained G3 and G4 toxicity (Common Terminology Criteria for Adverse Events versions 4.0 and 5) and the side effects conditioning a subsequent cycle delay.

The response assessment was defined with morphologic diagnostic testing (MRI or CT) every 3 mo for the first 2 y then every 6 mo until progression. Response Assessment in Neuro-Oncology criteria were used to assess the radiologic response (24). Functional ^111^In-octreoscan or ^68^Ga-DOTA-peptide PET/CT imaging was performed 6 mo after PRRT and then was performed once a year.

### Statistical Analysis

Overall data were summarized by median and range for continuous variables and by frequencies and percentage for categoric variables.

Disease control rate (DCR) was defined as the percentage of patients who achieved complete response, partial response, or stable disease at the end of PRRT. A Fisher exact test was used to evaluate the association between DCR and patients’ clinical features. DCRs were reported along with 95% CIs.

Progression-free survival (PFS) was defined as the time from the start of PRRT to the date of progression or death or last tumor evaluation, whereas overall survival (OS) was considered the time from the start of PRRT to the time of death or last follow-up. The Kaplan–Meier method and log-rank test were used to compare PFS and OS among groups of patients. The median PFS and OS values and the corresponding 95% CIs were reported.

All *P* values were 2-sided, and *P* values of less than 0.05 were considered statistically significant. Analyses were performed with STATA 15.1 statistical software (StataCorp). Full details can be found in the trial protocols on ClinicalTrials.gov (protocol number: IRST 100 01; eudract number: 2007-005517-20; protocol number: IRST 100.06; eudract number: 2011-002891-18; eudract number: 2012-003155-11; protocol number: irst100.26; eudract number: 2015-004727-31).

## RESULTS

We retrieved data from 42 patients, of whom 20 were women and 22 men (median age, 61 y). The median follow-up was 63 mo. Of the 39 cases of histologically confirmed meningiomas for which the grading was available, 11 (28%) were G1, 24 (62%) were G2, and 4 (10%) were G3. Of the 42 patients, 40 (95%) had at least 1 surgery and 33 (79%) had at least 1 radiotherapy treatment. Neurologic symptoms were exhibited by 34 patients (81%), whereas 32 patients (76%) were on anticonvulsant or antiedema drug therapy. Four patients (10%) received 1 line of chemotherapy, and 3 of them underwent off-label somatostatin analog therapy (25). Eastern Cooperative Oncology Group PS was 0 in 20 patients (48%), 1 in 14 patients (33%), and 2 in 8 patients (19%).

According to the SSTR2 PET images, 7 patients (17%) had a lesion activity higher than that in the liver and were classified as G3 according to the Krenning scale, whereas the other 35 (83%) patients had an activity higher than that in the spleen and were classified as G4. The tumor burden was considered moderate in 16 patients (38%) and extended in the remaining 26 patients (62%). Table 1 reports the distribution of patient characteristics.

**TABLE 1.** Patient Characteristics

Parameter	Total *n* = 42
Sex	
Female	20 (48)
Male	22 (52)
Age at therapy (y)	64 (31–86)
Histologic grade	
G1	11 (28)
G2	24 (62)
G3	4 (10)
Missing	3
Surgery	
No surgeries	2 (5)
1	15 (36)
2	8 (19)
≥3	17 (40)
Radiotherapy cycles	
No radiotherapy	9 (21)
1	18 (43)
2	11 (26)
≥3	4 (10)
^68^Ga-PET uptake	
3	7 (17)
4	35 (83)
Disease extension	
Moderate	16 (38)
Extended	26 (62)
ECOG PS	
0	20 (48)
1	14 (33)
2	8 (19)
Neurologic symptoms	
No	8 (19)
Yes	34 (81)
Radiopharmaceutical	
^ 90^Y	5 (12)
^ 177^Lu	37 (88)
PRRT cycles	
<4	15 (36)
≥4	27 (64)

ECOG = Eastern Cooperative Oncology Group.

Qualitative data are number and percentage. Continuous data are median and range.

**FIGURE 2.**
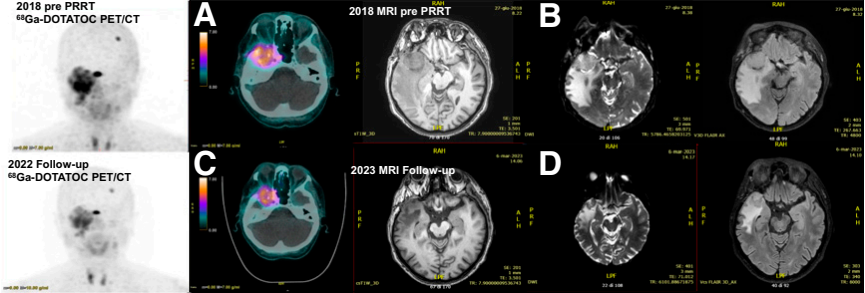
(A) ^68^Ga-DOTATOC PET/CT–positive image of symptomatic patient who underwent 2 resections in 1990 and in 1992. (B) MRI from 2018 of same patient with recurrent and progressive meningioma, who also received radiotherapy (50 Gy/25 fractions) with neither disease nor symptom improvement. After multidisciplinary discussion, decision was made to include patient in our ^177^Lu protocol, and after 5 cycles of ^177^Lu-DOTATATE PRRT, patient obtained partial response with clinical improvement. ^68^Ga-DOTATOC PET/CT performed in 2022 (C) showed persistent positivity and partial response that was confirmed at last MRI on June 3, 2023 (D).

Five patients were treated with ^90^Y-DOTATOC with a median cumulative activity was 11.1 GBq (range, 6.6–11.4 GBq) administered in a median number of 5 cycles (range, 3–6 cycles). In the remaining 37 patients treated with ^177^Lu-DOTATATE, the median cumulative activity was 22 GBq (range, 9.2–33 GBq) administered in a median number of 4 cycles (range, 2–6 cycles) (Fig. 2).

No complete responses were seen. One patient (2%) obtained a partial response, and 23 patients (55%) had stable disease. The overall DCR was 57% (95% CI, 42%–71%) with a median duration of response of 12 mo (95% CI, 8.3–47.7 mo). Progressive disease was seen in 18 patients (43%).

No statistically significant differences were seen in DCR according to age (64 or younger vs. older than 64), grading (G1 vs. G2 vs. G3), tumor burden (moderate vs. extended), previous treatments, and PS (Table 2).

**TABLE 2.** DCR After PRRT

Parameter	DCR*	*P*
Overall	0.57 (0.42–0.71)	
Sex		
Female	0.60 (0.37–0.79)	0.72
Male	0.55 (0.34–0.74)	
Age at therapy (y)		
≤64	0.71 (0.50–0.86)	0.06
>64	0.39 (0.19–0.63)	
Histologic grade		
G1	0.64 (0.33–0.86)	0.50
G2–G3	0.50 (0.32–0.68)	
^68^Ga-PET uptake		
3	0.57 (0.22–0.86)	1
4	0.57 (0.40–0.73)	
Surgery		
≤1	0.71 (0.45–0.88)	0.21
≥2	0.48 (0.29–0.67)	
Radiotherapy cycles		
≤1	0.59 (0.40–0.76)	0.75
≥2	0.53 (0.29–0.76)	
Concomitant therapy		
No	0.80 (0.45–0.95)	0.15
Yes	0.50 (0.33–0.67)	
Disease extension		
Moderate	0.75 (0.48–0.91)	0.10
Extended	0.46 (0.27–0.66)	
ECOG PS		
0	0.65 (0.42–0.83)	0.37
1–2	0.50 (0.30–0.70)	
Neurologic symptoms		
No	0.63 (0.28–0.88)	1
Yes	0.56 (0.38–0.72)	

* 95% CI in parentheses.

ECOG = Eastern Cooperative Oncology Group.

**FIGURE 3.**
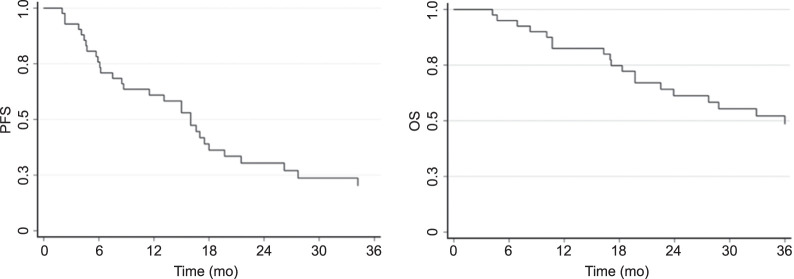
Kaplan–Meier curve of PFS (left) and OS (right) of cohort.

The median progression free survival (mPFS) was 16 mo (95% CI, 8.5–19.7 mo), and the median overall survival (mOS) was 36 mo (95% CI, 22.5–65.1 mo). Figure 3 shows the Kaplan–Meier curves for both PFS and OS.

These positive findings were confirmed in the largest subgroup composed of 37 patients treated with ^177^Lu-DOTATATE (62% did at least 4 cycles). In this group, with a median follow-up of 46.7 mo (95% CI, 31.7–70.7 mo), the DCR was 54% (95% CI, 38%–70%), mPFS was 15 mo (95% CI, 7.5–17.5 mo), and mOS was 33 mo (95% CI, 19.7–65.1 mo).

These results were seen across the subgroups analyzed (age, tumor burden, Eastern Cooperative Oncology Group PS). PFS was statistically different according to histology grade and previous surgical procedures. G1 patients showed a mPFS of 27.7 mo (95% CI, 4.4 mo to not recorded) compared with 11.5 mo (95% CI, 5.9–16 mo) for G2 and G3 patients (*P* = 0.007). mPFS was 27.7 mo (95% CI, 15–46.5 mo) for patients who underwent less than 2 surgeries compared with 11.5 mo (95% CI, 4.6–16 mo) for patients who underwent more than 2 surgeries before PRRT (*P* = 0.002). Concerning OS, patients who underwent less than 2 surgeries had a mOS of 65.1 mo (95% CI, 22.5 mo to not recorded) compared with 27.7 mo (95% CI, 17.1–44 mo) for patients who underwent more than 2 surgeries before PRRT (*P* = 0.04).

In those patients who received previous radiotherapy, PRRT seems not to be detrimental. Indeed, differences in mPFS (<2 cycles in 17 mo, 95% CI, 6.2–27.7 mo vs. ≥2 cycles in 15 mo, 95% CI, 4.1–17.5 mo) and mOS (<2 cycles in 43.3 mo, 95% CI, 17–103.8 vs. ≥2 cycles in 27.7 mo, 95% CI, 18.3–44 mo) were not significant (*P* = 0.15 and *P* = 0.40, respectively). Table 3 shows all of the results for PFS and OS analysis, whereas the supplemental materials provide a detailed description (supplemental materials are available at http://jnm.snmjournals.org).

**TABLE 3.** mPFS and mOS from Start of PRRT

Parameter	mPFS (mo)*	*P*	mOS (mo)*	*P*
Overall	16 (8.5–19.7)		36 (22.5–65.1)	
Sex				
Female	16 (5.9–39.2)	0.38	36 (17–71)	0.43
Male	16 (6.1–18)		42.4 (18.3–NR)	
Age at therapy (y)				
≤64	17.5 (6.2–26.2)	0.55	65.1 (19.7–103.8)	0.31
>64	16 (4.6–21.5)		27.7 (17–43.3)	
Histologic grade				
G1	27.7 (4.4–NR)	0.007	43.3 (4.2–NR)	0.32
G2–G3	11.5 (5.9–16)		32.9 (18.3–44)	
^68^Ga-PET uptake				
3	6.2 (4.1–NR)	0.46	NR (4.7–NR)	0.23
4	16 (8.7–21.5)		36 (19.7–65.1)	
Surgery				
≤1	27.7 (15–46.5)	0.002	65.1 (22.5–NR)	0.04
≥2	11.5 (4.6–16)		27.7 (17.1–44)	
Radiotherapy cycles				
≤1	17 (6.2–27.7)	0.15	43.3 (17–103.8)	0.40
≥2	15 (4.1–17.5)		27.7 (18.3–44)	
Concomitant therapy				
No	17.5 (4.4–21.5)	0.76	NR (10.1–NR)	0.20
Yes	15 (6.2–26.2)		32.9 (18.3–65.1)	
Disease extension				
Moderate	17.5 (13.1–34.2)	0.43	42.4 (17.1–103.8)	0.90
Extended	8.7 (4.7–19.7)		32.9 (22.5–71)	
ECOG PS				
0	16.6 (5.7–26.2)	0.95	71 (22.5–NR)	0.10
1–2	15 (7.5–27.7)		27.7 (16.3–44)	
Neurologic symptoms				
No	6.1 (3.8–NR)	0.37	NR (10.7–NR)	0.34
Yes	16.6 (11.5–21.5)		36 (22.5–65.1)	

* 95% CI in parentheses.

ECOG = Eastern Cooperative Oncology Group; NR = not reached.

### Retreatment

Six patients with persistent ^68^Ga-DOTATOC PET/CT positivity were retreated with ^177^Lu-DOTATATE, 3 after previous ^90^Y-DOTATOC (median cumulative activity of 11.1 GBq for ^90^Y-DOTATOC at first PRRT) and 3 after ^177^Lu-DOTATATE (median cumulative activity of 16.5 GBq for ^177^Lu-DOTATATE at first PRRT). The median injected dosage of ^177^Lu-DOTATATE was 13 GBq for a median of 4 cycles (range, 2–6 cycles). From the start of the retreatment PRRT, the median follow-up period was 75.8 mo (95% CI, 12.4 mo to not recorded), the mPFS was 6.5 mo (95% CI, 3.6 mo to not recorded), and the mOS was 17 mo (95% CI, 5.5 mo to not available).

### Toxicity

In our series, the treatments were well tolerated by the patients, and there were no cases of symptomatic worsening of patient conditions due to early or late toxicity. Only 1 patient had grade 3 platelet toxicity, which persisted at the time of subsequent treatment, causing therapy delay first and subsequent therapy cessation. Of the 6 retreated patients, 4 had persistent G1 anemia as side effect, whereas 1 had transient G2 toxicity for neutrophils.

## DISCUSSION

Meningiomas, commonly detected in clinical practice, present a significant clinical management problem because of the high number of symptomatic patients and frequent recurrence of the tumor despite surgical treatments and primary or second-line radiotherapy (1*,*^2^).

There are still certain unanswered questions about the management of meningiomas, which highlights the need for fresh therapeutic opportunities. One such opportunity is represented by the predominant SSTR2 expression, which can be used by labeling somatostatin analogs with ^68^Ga for PET/CT diagnosis and β-emitting radiopharmaceuticals for therapy.

Of 42 patients enrolled, 5 were treated with ^90^Y-DOTATOC and 37 with ^177^Lu-DOTATATE. All patients were in a progressive status and had a positive SSTR2 scintigraphy obtained with ^111^In-octreoscan in some of the first enrolled patients and with ^68^Ga-DOTATOC PET/CT for the large majority. The uptake intensity was evaluated semiquantitatively in a double-masked procedure by 2 nuclear medicine expert according to the Krenning score which could be used for ^68^Ga-PET/CT images. To guarantee the proper tumor irradiation, only patients with grades 3 and 4 tumors have been designated for therapy. The significant proportion of patients (83%) who had 4° uptake included in the present study could condition the efficacy of the treatment and could be considered as a limitation.

There are currently few studies reported treating recurrent meningiomas; these studies have small cohorts and do not always indicate the number of cycles, doses, or the standard radiopharmaceutical used. In a previously published study comprising 29 patients who received 5–15 GBq of ^90^Y-DOTATOC, a disease stabilization was observed in 66% of the patients and the mOS was 40 mo (16). A metaanalysis regarding 111 patients with treatment-refractory meningiomas considered performing PRRT with a radiopharmaceutical based on either ^90^Y or ^177^Lu, with different DOTA-peptide and cumulative dosages (21). This study documented a 63% DCR, a 6-mo PFS, and a 12-mo OS. Both PFS and OS were correlated with tumor grade: 94% and 88% for G1, 48% and 71% for G2, and 0% and 52% for G3, respectively.

The patients involved in the present study exhibit a significant frequency of certain features linked to a poor prognosis when compared with the population of previous studies that have already been published. These findings merit additional discussion. We had a high proportion of G2 and G3 patients (72%), a high disease burden in 62% of cases, specific symptoms in 81% of patients, and a high rate of previous cytotoxic therapy (76% of cases), beginning with high levels of radiation and surgery as pretreatments (Table 1). These data indicate that all patients are in an advanced stage of disease and that their overall clinical state is progressing.

Despite the high frequency of negative prognostic characteristics in the patients, the overall results obtained for DCR, mPFS, and mOS are representative of a good treatment activity that offers in an orphan pathology a well-tolerated and effective tool (Tables 2 and 3).

Unfortunately, because of the study design, we did not have a control group. With well-known adverse effects typical of antiangiogenic medications, the mOS for mixed grades II/III meningioma in a recently published trial using bevacizumab was 24 mo. If we look to our data, GII/III meningioma patients obtained a mOS of 32.9 mo (range, 18.3–44 mo) with a good toxicity profile (26).

Data are confirmed from the active and effective results obtained in the specific ^177^Lu-DOTATATE group of 37 patients with advanced disease despite only 62% of them completing at least 4 cycles. This group first confirmed the treatment homogeneity that the patient had with the different protocols with a mean injected activity of 22 GBq in 4 cycles and then showed activity and efficacy results similar to the best data already reported with ^90^Y-DOTATOC (16*,*^17^).

A significantly better result was obtained in patients with a lower tumor grade and only 2 previous surgeries, but in general, the higher efficacy seen in younger people, G1, less extensive tumors, better well performing and asymptomatic patients, albeit not statistically significant, suggests a reasonably better response in earlier stages of the disease.

Retreatment PRRT in relapsed patients is an interesting option also in very advanced situations when blood tests are permissive and there is a persistent positivity on ^68^Ga-DOTA-peptide PET/CT lesions. This retreatment PRRT is well tolerated by patients and offers the opportunity to obtain lesion stabilization, although in a less effective way.

It is also intriguing to investigate the extremely low reported toxicity. We can first explain it by the minimal dosages used and the absence of visceral disease. Our research confirms the commonly acknowledged acceptable tolerability up to dosages of 29.6 GBq, the cumulative activity provided with the registered Lutathera (Novartis). In this work, we additionally highlight the well-tolerated PRRT retreatment to more than 37 GBq of ^177^Lu-DOTATATE, as evidenced by the lack of renal and bone marrow toxicity as well as the absence of neurologic impairment. Actually, despite advancements in technology, the main limit to additional radiotherapy courses remains the risk of brain toxicity.

To the best of our knowledge, this is the largest prospective study on PRRT in patients with meningiomas with pending results of some prospective trials testing the use of ^177^Lu- and ^90^Y- PRRT (NCT03971461, NCT04082520, NCT03273712).

The development of dedicated clinical trials should be encouraged (27).

## CONCLUSION

Our data from patients with advanced refractory meningiomas, collected in a consecutive way for more than 10 y, confirmed PRRT tolerability, highlighted a good effectiveness, and suggested its potential improvement if applied in an earlier stage. Further studies are encouraged to confirm the efficacy of salvage PRRT in meningiomas, a well-tolerated therapy that may extend disease control. Because of the preliminary data reported here, in patients with persistent ^68^Ga-PET/CT positivity, retreatment with PRRT should be investigated in a larger study. The hypothetical incremental efficacy of the association of radiotherapy and PRRT have promising results and deserves further studies.

## DISCLOSURE

This work was partly supported by contributions from Ricerca Corrente by the Italian Ministry of Health, innovative therapies, phase I–III clinical trials, and therapeutic strategy trials based on preclinical models, oncoimmunologic mechanisms and nanovectors, and AIRC. No other potential conflict of interest relevant to this article was reported.

## Notes

KEY POINTS **QUESTION:** For patients with recurrent and refractory meningiomas, is PRRT an effective option? **PERTINENT FINDINGS:** Heavily pretreated meningioma patients with positive ^68^Ga-PET/CT scans treated with ^177^Lu-DOTATATE and ^90^Y-DOTATOC obtained a DCR of 57% with an optimal safety profile. **IMPLICATIONS FOR PATIENT CARE:** PRRT in heavily pretreated and progressing meningioma patients overexpressing SSTR2 showed good disease control, making this a treatment option for this orphan disease.
